# Supplementary material for: A transcriptional activator effector of Ustilago maydis regulates hyperplasia in maize during pathogen-induced tumor formation
Source: Nat Commun. 2023 Oct 23;14:6722. doi: 10.1038/s41467-023-42522-w (PMC10593772; doi:10.1038/s41467-023-42522-w)
Supplement: Supplementary file 1 — Supplementary Information [file 41467_2023_42522_MOESM1_ESM.pdf]

Supplementary Information:

Supplementary Fig. 1-6; Supplementary Tables 1-3, Extended figure legend

A transcriptional activator effector of *Ustilago maydis* regulates hyperplasia in maize during pathogen-induced tumor formation

Weiliang Zuo[1*], Jasper R. L. Depotter[1,4], Sara Christina Stolze[2], Hirofumi Nakagami[2,3], Gunther Doehlemann[1*]

1: Institute for Plant Sciences and Cluster of Excellence on Plant Sciences (CEPLAS), University of Cologne, Cologne 50674, Germany

2: Protein Mass Spectrometry, Max-Planck Institute for Plant Breeding Research, Carl-von-Linné Weg 10, 50829 Cologne, Germany

3: Basic Immune System of Plants, Max Planck Institute for Plant Breeding Research, Cologne 50829, Germany
4: Current address: Bioinformatics and Biostatistics, The Francis Crick Institute, 1 Midland Road, London, NW1 1AT, UK

***Correspondence to:** g.doehlemann@uni-koeln.de; wzuo@uni-koeln.de

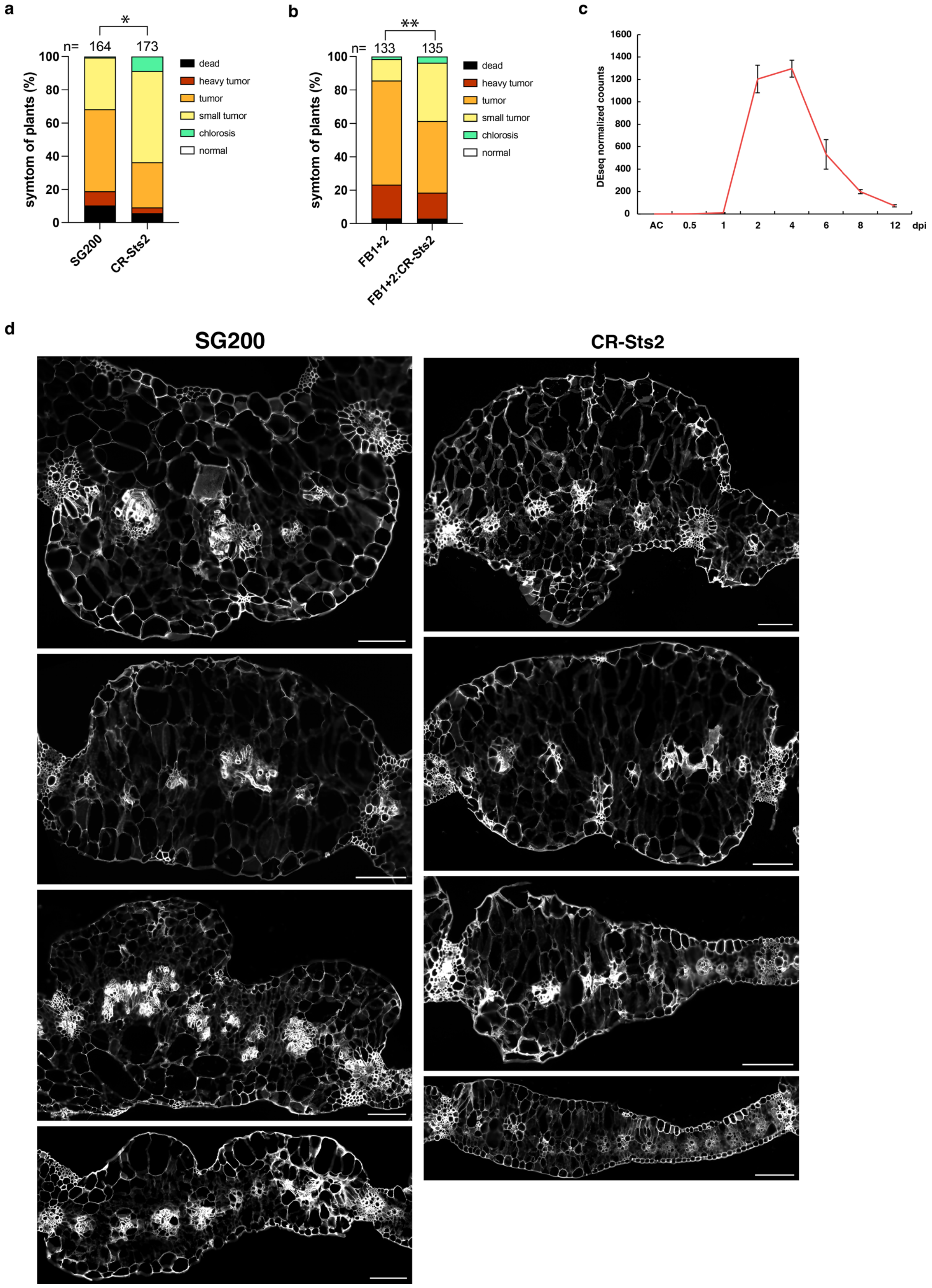

Supplementary Fig1. Sts2 is induced during *U. maydis* infection and regulates hyperplasia tumor formation. a, Disease symptoms of SG200, CR-Sts2 on maize cultivar Early Golden

Bantam. N is the total number of plants infected from 4 independent infections. * $p<0.05$. The Student's *t-test* was used for statistic test. **b,** Disease symptoms of *U. maydis* wildtype FB1+2 and their derived CR-Sts2 mutants on maize cultivar Golden Bantam. N is the total number of plants infected from three independent infection experiments. ** $p<0.01$. The Student's *t-test* was used for statistic test. **c,** Expression of Sts2 in *U. maydis* FB1×FB2 during the whole biotrophic infection from published data. **d,** More microscope photos of transverse section from SG200 and CR-Sts2 at 12 dpi on maize cultivar Golden Bantam. Each photo represents the typical phenotype from individual plant.

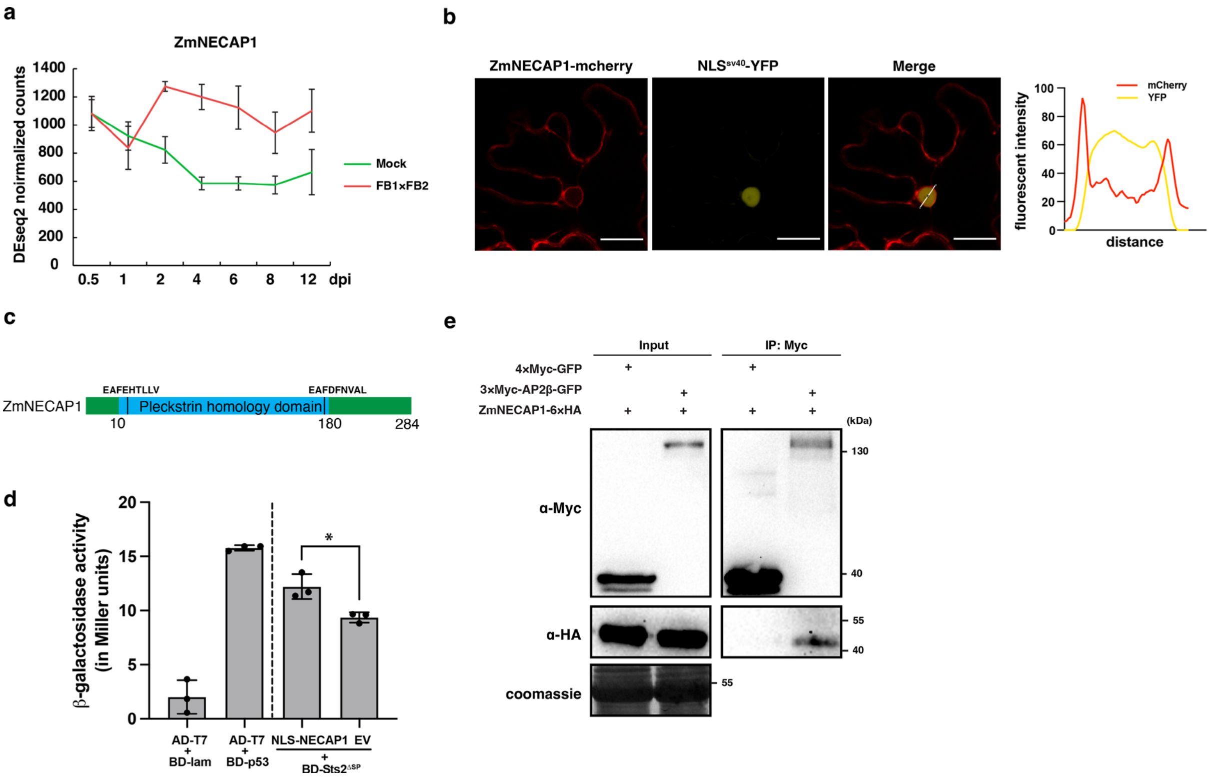

Supplementary Fig2. ZmNECAP1 is a novel plant transcriptional activator induced during *U. maydis* infection. a, Expression of ZmNECAP1 is induced during *U. maydis* FB1×FB2 infection. Expression data taken from Lanver et al., 2017 [(11)] **b,** Subcellular localization of ZmNECAP-mCherry and NLS^{SV40}-YFP in N. *N. Benthamiana.* The YFP and mCherry intensities were measured from the solid white line as shown in the "Merge". Scale bar = 20 µm. **c,** Domain arrangement of ZmNECAP1. Two vertical lines indicate the position of the separated two TADs and the amnio acid sequences are shown above. **d,** β-galactosidase activity of AH109 strains transformed with BD-$Sts2^{\Delta SP}$/NLS^{Gal4}-ZmNECAP1 and BD-$Sts2^{\Delta SP}$/EV. BD-lam/AD-T7 and BD-p53/AD-T7 strains are used as negative and positive controls, respectively. Student's *t-test* was used for significance test. * $p<0.05$. **e,** Co-IP western blot shows the interaction of ZmAP2β and ZmNECAP1. Around 1.38% of total extract from each sample are shown as input.

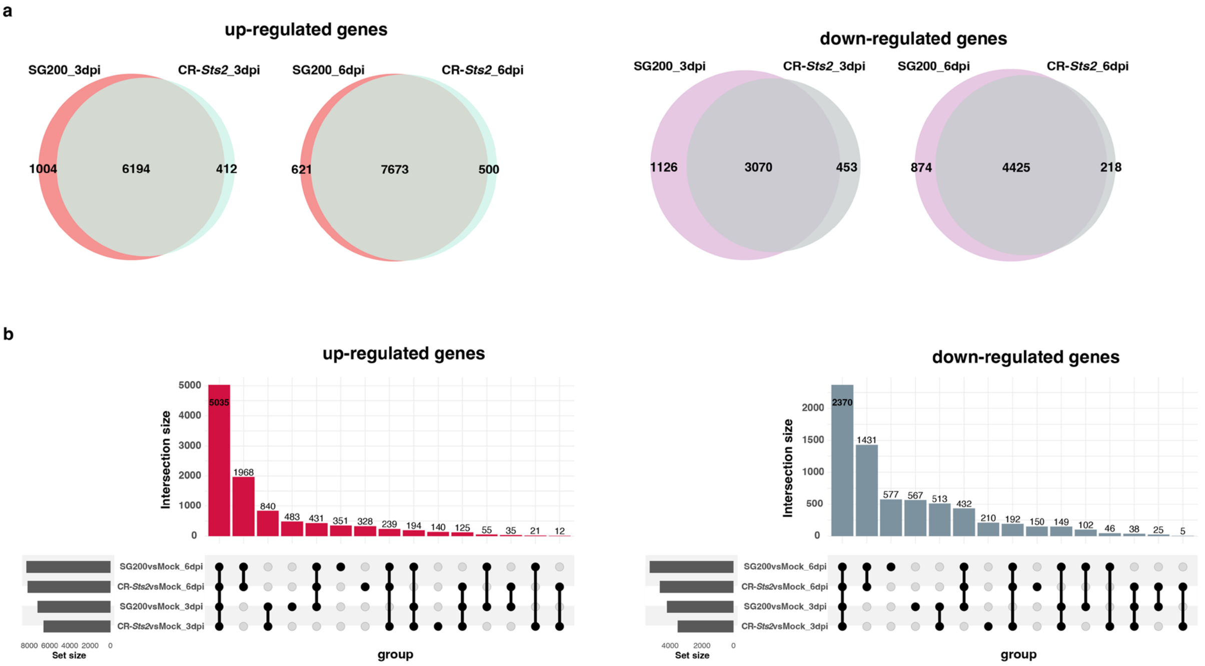

Supplementary Fig3. Differentially expressed genes compared to Mock. a, The Venn diagrams show the numbers of differentially expression genes between SG200vs. Mock and CR-Sts2 vs. Mock at 3 and 6 dpi, respectively. **b,** The intersection diagrams show the genes numbers in different paired comparison.

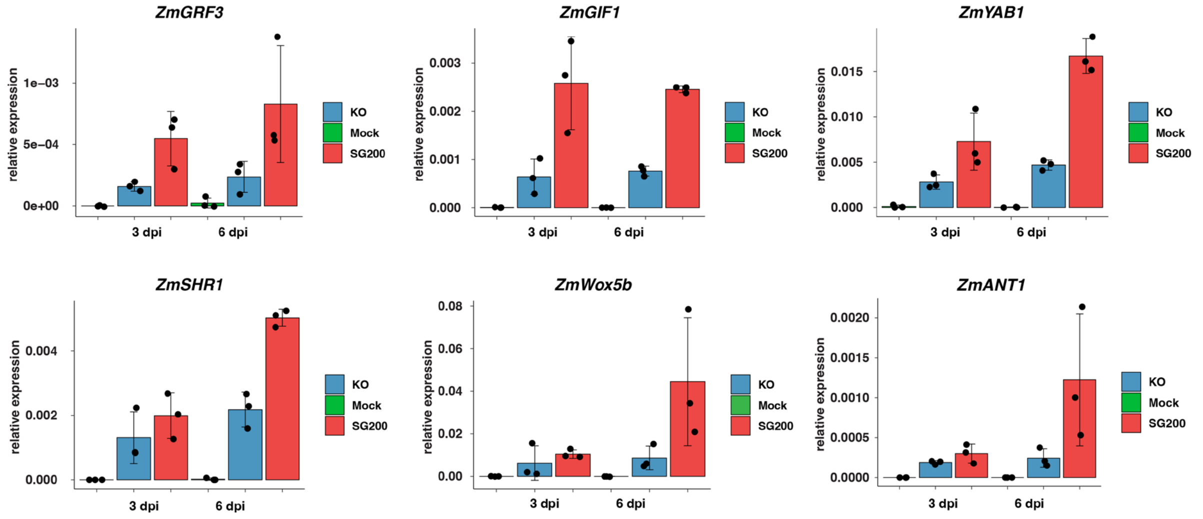

Supplementary Fig4. Expression of maize genes upon *U. maydis* infection in RNA-seq samples by qPCR. Expression levels of leaf developmental regulators detected by qPCR in RNA-seq samples. The value of $2^{-\Delta Ct}$ between gene of interest (GOI) and *ZmGAPDH* are calculated and plotted.

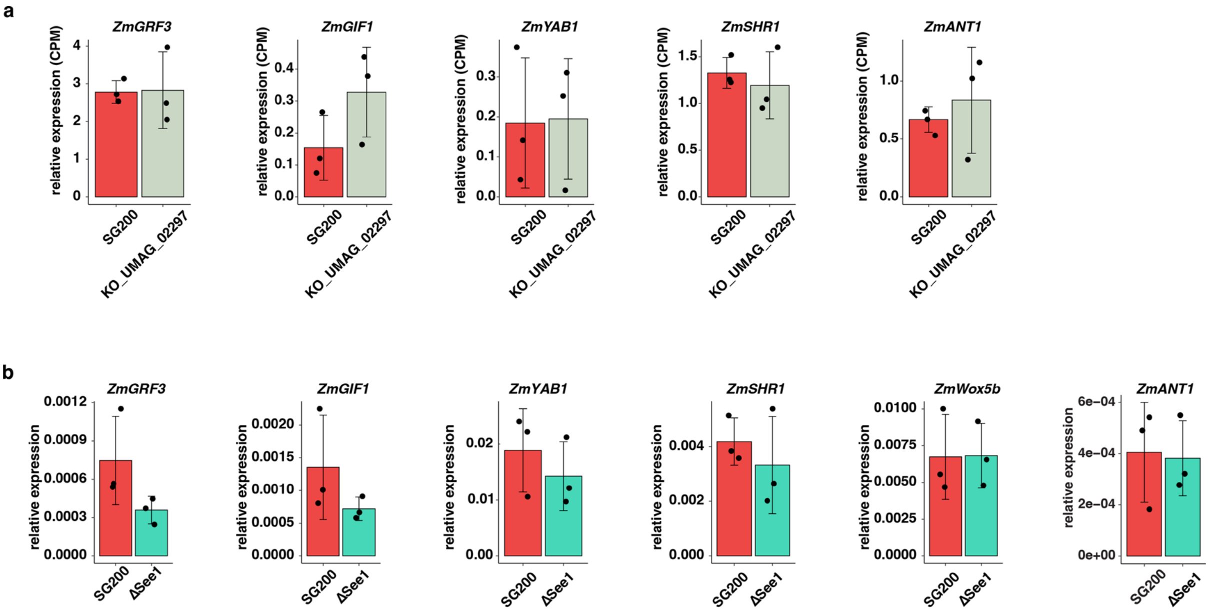

Supplementary Fig5. Expression of maize genes upon different *U. maydis* effector mutants' infection. a, Relative expression levels of maize leaf developmental regulators upon SG200 and KO_UMAG_02297 infection in maize line CML322 at 3 dpi from published data[39]. The CPM (counts per million) are plotted. **b,** Expression of leaf developmental regulators in SG200 and ΔSee1 mutant infected Golden Bantam at 6dpi by qPCR. The values of $2^{-\Delta Ct}$ between GOI and *ZmGAPDH* are calculated and plotted.

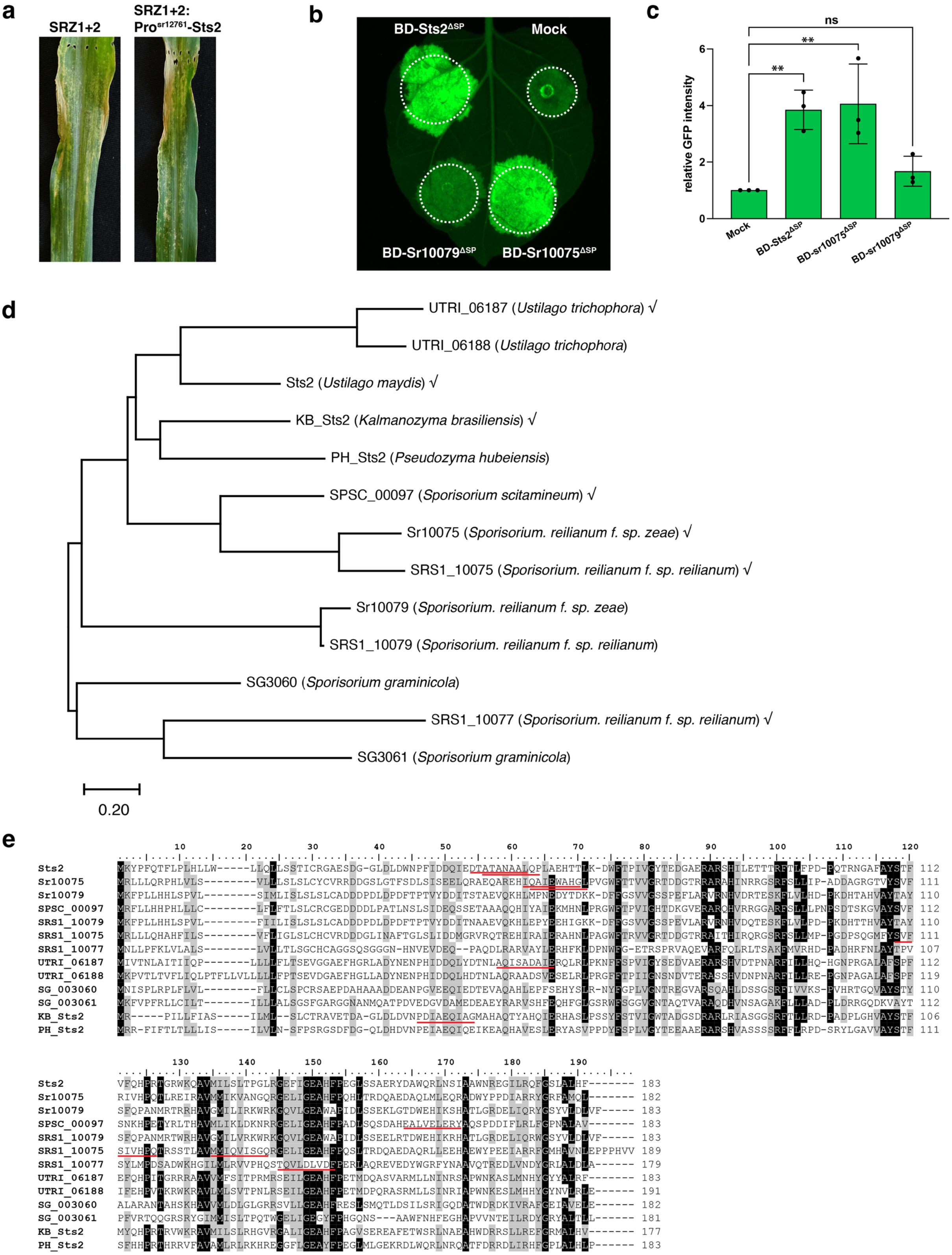

Supplementary Fig6. Sts2 orthologs in smut fungi. a, Photos show leaves infected by *S. reilianum* and *S. reilianum* overexpressing Sts2. The photos were taken at 7 dpi. **b,** BD-Sr10075$^{\Delta SP}$ but not BD-Sr10079$^{\Delta SP}$ activates the Pro$^{5\times UAS\text{-}35Smini}$::GFP expression. The dashed circles indicate the infiltration area. **c,** Bar plot showing the GFP intensity from three biological replicates. Data shown are the mean value ± SD and normalized to the Mock. Dunnett's 1-way ANOVA test was used to determine the significance. ns, not significant, ** $p<0.01$. **d,**

Phylogenetic trees of Sts2 orthologs identified in *Ustilaginales* species. √: orthologs containing a predicted TAD. **e,** Sequence alignment of Sts2 orthologs identified in *Ustilaginales* species. The red lines show the TAD region in the protein sequences.

Supplementary Table 1. MS data

Supplementary Table 2. Gene ontology analysis of up- and down-regulated genes during *U. maydis* infection

Supplementary Table 3. Oligos sequencing used in the study

Extended Figure. Original images of western blots. a, the original western blot image of cell fractionation samples. **b,** the original Co-IP western blot image of ZmNECAP1 and Sts2. **c,** the original western blot image of BD-Sts2$^{\Delta SP}$ and its mutants from infiltrated *N. benthamiana* leaves. **d,** the original western blot image of BD-ZmNECAP1 and its mutant from infiltrated *N. benthamiana* leaves. **e,** the original Co-IP western blot image of ZmNECAP1 and ZmAP2β.
