# Supplementary material for: A transcriptional activator effector of Ustilago maydis regulates hyperplasia in maize during pathogen-induced tumor formation
Source: Nat Commun. 2023 Oct 23;14:6722. doi: 10.1038/s41467-023-42522-w (PMC10593772; doi:10.1038/s41467-023-42522-w)
Supplement: Supplementary file 3 — Description of Additional Supplementary Files [file 41467_2023_42522_MOESM3_ESM.pdf]

Description of Additional Supplementary Files

Supplementary Data 1. MS data

Supplementary Data 2. Gene ontology analysis of up- and down-regulated genes during *U. maydis* infection

Supplementary Data 3. Oligos sequencing used in the study
